# The impact of severe obesity on image quality and ventricular function assessment in echocardiography and cardiac MRI

**DOI:** 10.1007/s10554-024-03078-y

**Published:** 2024-04-16

**Authors:** Akash Goyal, Christopher D. Crabtree, Bryan C. Lee, Thura T. Harfi, Saurabh Rajpal, Vedat O. Yildiz, Orlando P. Simonetti, Matthew S. Tong

**Affiliations:** 1 Department of Internal Medicine, Division of Cardiovascular Medicine, The Ohio State University, 234 Davis Heart & Lung Research Institute, 473 W. 12th Avenue, Columbus, OH USA; 2 Department of Human Sciences, The Ohio State University, Columbus, OH USA; 3 OhioHealth Systems, Heart and Vascular Institute, Columbus, OH USA; 4 Center for Biostatistics, Department of Biomedical Informatics, The Ohio State University, Columbus, OH USA; 5 Department of Radiology, The Ohio State University, Columbus, OH USA; 6 Davis Heart & Lung Research Institute, 473 W. 12th Avenue, Columbus, OH USA

**Keywords:** Cardiac MRI, Echocardiography, Image quality, Severe obesity

## Abstract

This study sought to evaluate the impact of severe obesity on image quality and ventricular function assessment in cardiovascular magnetic resonance (MRI) and trans-thoracic echocardiography (TTE). We studied 100 consecutive patients who underwent clinically indicated cardiac MRI and TTE studies within 12 months between July 2017 and December 2020; 50 (28 females and 22 males; 54.5 ± 18.7 years) with normal body mass index (BMI) (18.5–25 kg/m^2^) and 50 (21 females and 29 males; 47.2 ± 13.3 years) with severe obesity (BMI ≥ 40 kg/m^2^). MRI and TTE image quality scores were compared within and across cohorts using a linear mixed model. Categorical left (LVF) and right (RVF) ventricular function were compared using Cohens Kappa statistic. Mean BMI for normal weight and obese cohorts were 22.2 ± 1.7 kg/m^2^ and 50.3 ± 5.9 kg/m^2^, respectively. Out of a possible 93 points, mean MRI image quality score was 91.5 ± 2.5 for patients with normal BMI, and 88.4 ± 5.5 for patients with severe obesity; least square (LS) mean difference 3.1, p = 0.460. TTE scores were 64.2 ± 13.6 for patients with normal BMI and 46.0 ± 12.9 for patients with severe obesity, LS mean difference 18.2, p < 0.001. Ventricular function agreement between modalities was worse in the obese cohort for both LVF (72% vs 80% agreement; kappa 0.53 vs 0.70, obese vs. normal BMI), and RVF (58% vs 72% agreement, kappa 0.18 vs 0.34, obese vs. normal BMI). Severe obesity had limited impact on cardiac MRI image quality, while obesity significantly degraded TTE image quality and ventricular function agreement with MRI.

**Supplementary Information:**

The online version contains supplementary material available at 10.1007/s10554-024-03078-y.

## Introduction

Obesity affects over 40% of adults in the United States with rising prevalence and worsening severity; nearly 10% of adults now have a body mass index (BMI) ≥ 40 kg/m^2^ (severe or Class III obesity) [1, 2]. Obesity has long been recognized as a strong independent predictor of cardiovascular disease (CVD) [3–5], and non-invasive imaging modalities including transthoracic echocardiography (TTE), computed tomography (CT), single photon emission tomography (SPECT), and cardiovascular magnetic resonance imaging (MRI) are commonly used to evaluate CVD. However, imaging of patients with severe obesity poses technical challenges for all modalities, including table weight and bore diameter limitations, higher radiation dosing, and signal attenuation that reduces signal–noise ratio (SNR) and causes artifacts [6–9].

The accuracy of left (LVF) and right (RVF) ventricular function assessment is critical for contemporary management of numerous cardiovascular diseases [10]. MRI has high spatial and temporal resolution, measures chamber volumes without geometric assumptions, and is the reference standard for quantitative LVF and RVF [11]. Though inter-observer variability is improved with three-dimensional contrast-enhanced TTE, the limits of TTE agreement with cardiac MRI remains wide for both LVF [12] and RVF [13, 14]. The potential for patient misclassification based on inaccurate ventricular function assessment can subsequently lead to additional downstream testing or unnecessary procedures.

While TTE is often used to evaluate for a cardiac etiology of symptoms such as dyspnea or extremity edema, image quality can be severely hampered in patients with obesity, leading to diagnostic uncertainty [7, 15]. CT and SPECT are used to evaluate coronary artery disease but do not provide the hemodynamic information necessary for a complete evaluation of CVD. Furthermore, in patients with high BMI, CT and SPECT require higher contrast loads and radiation doses, and attenuation artifacts can be problematic [8, 9]. Given these limitations, the optimal cardiac imaging strategy in patients with severe obesity remains unclear.

Though the negative impact of severe obesity on TTE image quality is well recognized, this is less well understood for cardiac MRI, and the effect on ventricular function assessment has not been previously studied. Emerging data has demonstrated the diagnostic utility of cardiac MRI in severe obesity [16, 17]. However, there has been no direct comparison of image quality or ventricular function between severely obese and normal weight cohorts across cardiac MRI and TTE. In this study, we hypothesize that severe obesity adversely affects MRI image quality to a lesser degree than TTE. Additionally, we hypothesize that LVF and RVF agreement between TTE and MRI is worse in patients with severe obesity compared with patients with normal BMI.

## Materials and methods

### Study cohort and design

A total of 134 patients with normal BMI and 143 patients with severe obesity were consecutively screened from patients who had clinical cardiac MRI and TTE studies between July 2017 and December 2020. Exclusion criteria included: greater than 12 months between index MRI and TTE and limited TTE or MRI studies. General MRI exclusion criteria were known hypersensitivity to gadolinium-based contrast agents, glomerular filtration rate < 30 mL/min/1.73 m^2^, pregnancy, and hemodynamic instability. Image quality was scored and ventricular volumes were compared. Of the final cohort, no patients had a clinically reported non-diagnostic TTE or cardiac MRI. Additionally, no patients were referred for cardiac MRI with a stated indication of non-diagnostic TTE. This retrospective HIPAA-compliant study was approved by the local Institutional Review Board with waiver of informed consent (IRB #2020H062).

### Image quality scoring

**Fig. 1:**
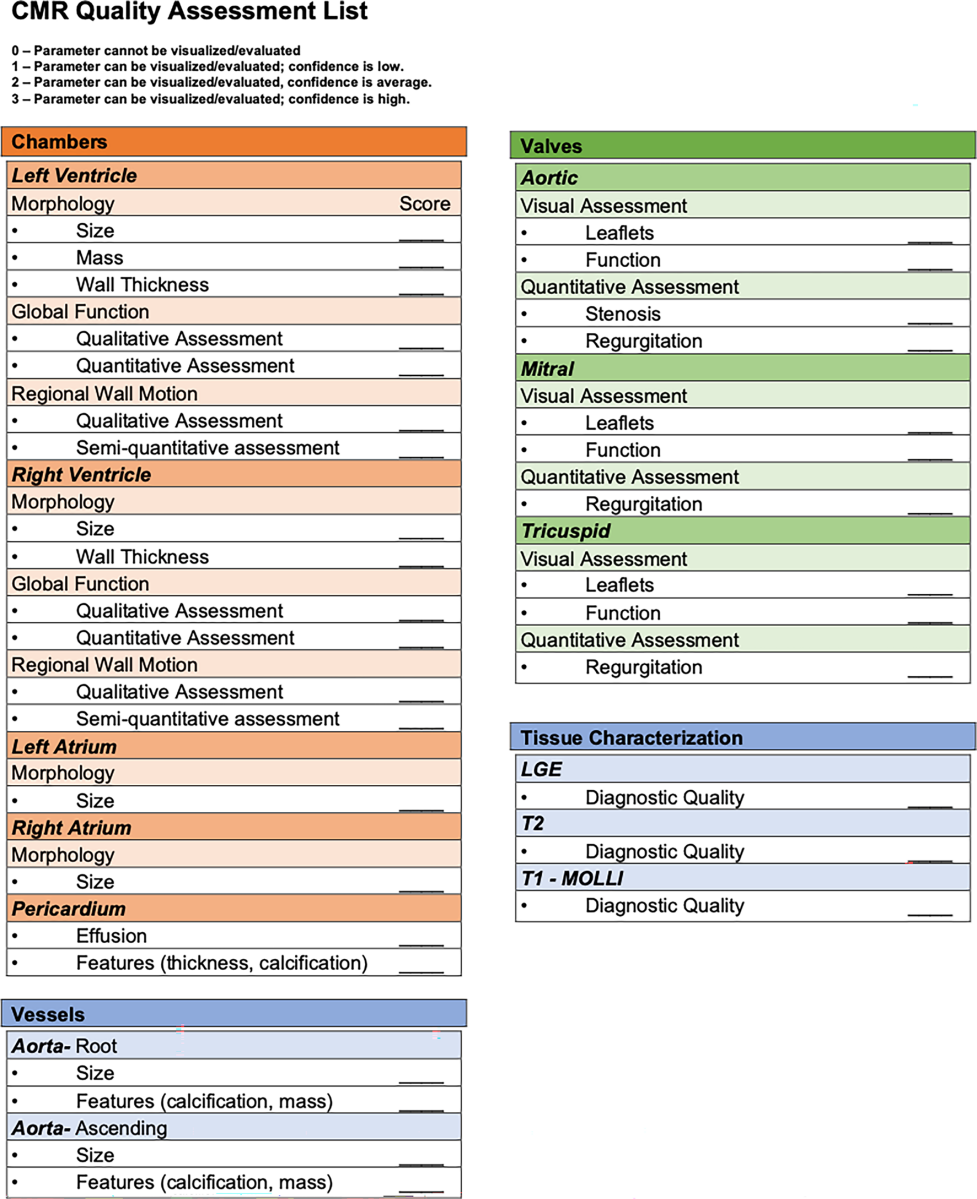
Cardiac MRI image quality assessment list. Image quality scoresheet used for cardiac MRI outlining the 31-part list of routine imaging categories. Higher scores indicate higher level of quality for a maximum total possible score of 93 points. Note the additional, separately-scored tissue characterization for MRI

**Fig. 2:**
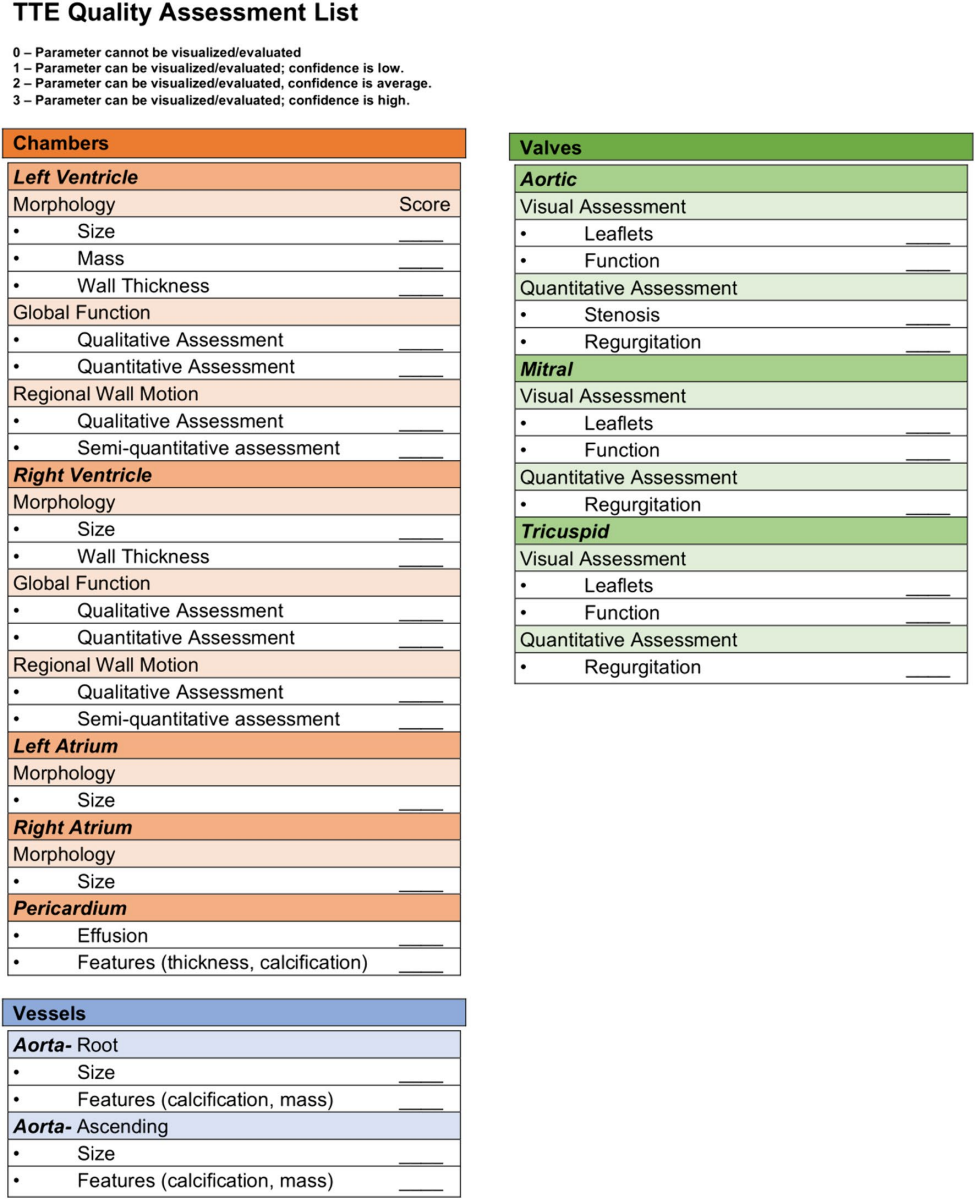
TTE image quality assessment list. Image quality scoresheet used for TTE outlining the 31-part list of routine imaging categories. Higher scores indicate higher level of quality for a maximum total possible score of 93 points

To compare the quality of MRI and TTE images, we developed a 31-part list of imaging categories (Figs. 1, 2) that could be applied equally to both modalities. Readers scored each parameter according to their level of confidence: parameter cannot be visualized/evaluated (Score 0); parameter can be visualized/evaluated with low (Score 1), average (Score 2), and high (Score 3) confidence, for a maximum total possible score of 93 points. Each part was designated into three commonly evaluated anatomical sections: chambers (left and right ventricles and atria), vessels (aortic segments), and valves (aortic, mitral, and tricuspid). Each section scored reader confidence in visual interpretation and quantitative measurements.

Image quality assessment for TTE was performed independently by two ACC/COCATS level-III TTE (TH, BL) readers with 16 and 8 years of experience, respectively, while image quality assessment for MRI was performed independently by two different ACC/COCATS level-III cardiac MRI (MT, SR) readers each with 10 years of experience. All readers were blinded to BMI and the other modality images. The pairs of readers for each modality first reviewed and scored 20 cases (not included in the study cohort) together to develop a consensus on image quality scoring definitions.

The image quality scores for MRI and TTE were tabulated independently by each reviewer. Every raw score for each structure (e.g., aortic valve, left atrium) was averaged between the two readers for both MRI and TTE, then summed to obtain a sub-total for each category (chambers, vessels, and valves), and summed again to obtain a total image quality score. A separate image quality score was assigned to MRI tissue characterization scans (T1 and T2 mapping, LGE) that have no TTE equivalent.

To quantify the proportion of patients with a potential clinical status change between MRI and TTE, we reviewed patients with LVF < 50% by either modality for discrepancies using LVF < 50% or < 35% as these are common cutoffs for changes in clinical management. A clinical status change was defined as any cardiovascular interventions performed between each imaging modality (e.g. revascularization, arrhythmia management, cardiac arrest, initiation of guideline directed therapy) that could affect ventricular function.

### Cardiovascular magnetic resonance imaging protocol

Cardiac MRI studies were performed using 1.5 T scanners with either 60 cm or 70 cm bore diameter: (MAGNETOM Avanto, Espree, Aera or Sola, Siemens Healthineers AG, Erlangen, Germany). Cardiac MRI images were acquired specific to the clinical indication following Society for Cardiovascular Magnetic Resonance recommendations [18]. All cardiac MRI studies were required to include 2-, 3-, and 4-chamber long-axis cine images, a complete short-axis cine stack, and at least one aortic or main pulmonary artery phase contrast scan without aliasing. Late gadolinium enhancement (LGE) and T1 and T2 maps were optional. No specific protocols were implemented for patients with obesity beyond standard sequence parameter modifications (i.e., larger field of view if wrap artifact present) to accommodate larger body habitus. Free-breathing real-time cine [19] was used in patients unable to breath hold. Oral benzodiazepines were administered as needed for claustrophobia. Quantitative LVF and RVF were measured by the clinical reader from contiguous short-axis cine images using validated software. LVF and RVF categorical groups were defined as follows: ejection fraction ≥ 50% = normal, 40–49% = mild, 30–39% = moderate, and ≤ 29% = severe dysfunction.

### Two-dimensional transthoracic echocardiography protocol

TTE studies were acquired by experienced sonographers using any of six clinical scanners: Vivid E95 and Vivid IQ (GE Vingmed Ultrasound, Horten, Norway), Acuson Bonsai and Acuson SC2000 (Siemens Healthineers AG, Erlangen, Germany), iE33 and Epiq (Philips Medical Systems, Andover, MA). Echocardiographic studies were required to meet the criteria for a complete TTE with Doppler per current American Society of Echocardiography (ASE) recommendations [20]. LV volumes were measured by biplane Simpson method from the apical 4- and 2-chamber views following ASE recommendations [21]. Microbubble contrast agent was administered (Definity®; Lantheus, North Billerica, MA, USA) if endocardial border definition was inadequate. LVF was assessed quantitatively and categorized using the same definitions as cardiac MRI. RVF was assessed categorically by visual assessment, tricuspid annulus excursion, S' tissue Doppler, and fractional area change. The final determination of LVF and RVF was at the discretion of the clinical readers, considering available visual and quantitative metrics.

### Statistical analyses

An unpaired t-test was used to compare continuous variables, while chi-square or Fisher exact test was used for proportions. Linear mixed model followed by either paired or unpaired t-tests were used to test primary and secondary hypotheses. Cohen’s kappa statistic was applied to test the agreement in LVF and RVF, defined as a patient having the same LVF or RVF categorical group for both MRI and TTE, using the following interpretation rubric: 0–0.20 = no agreement, 0.21–0.40 = fair agreement, 0.41–0.60 = moderate agreement, 0.61–0.80 = substantial agreement, and 0.81–1.00 = almost perfect agreement [22]. Wilcoxon sign rank test was used to compare MRI tissue characterization image quality between the cohorts. Polyserial correlations were used to determine the effect of time on categorical LVF and RVF differences.

## Results

### Clinical characteristics

Characteristics of the patients studied are shown in Table 1. After screening, the study cohort included 100 patients; 50 patients (age 54.5 ± 18.7 years; range: 22–92 years, 28 female) with normal BMI (22.2 ± 1.7 kg/m^2^) and 50 patients (age 47.2 ± 13.3 years; range: 20–73 years, 21 female) with severe (Class III) obesity (BMI 50.3 ± 5.9 kg/m^2^). Mean absolute time between MRI and TTE studies was 83.2 ± 87.8 days and was not statistically different between the cohorts (p = 0.845); 66% of the MRI and TTE pairs were within 90 days of each other. The most common indication for imaging was heart failure. The use of microbubble contrast for TTE was more frequent in the severely obese than the normal BMI cohort (64% vs 20%, p < 0.001). Mean LV ejection fraction was similar between the normal BMI and severely obese cohorts by MRI (45.5% ± 14.5% and 47.4% ± 17.6%, respectively, p = 0.558) and by TTE (46.0% ± 15.3% and 49.4% ± 16.3%, respectively, p = 0.283). The mean RV ejection fraction by cardiac MRI showed no significant difference between the normal BMI and severely obese cohorts (52% ± 11.5% and 47.8% ± 12.5%, respectively, p = 0.127). TTE similarly demonstrated no significant differences between normal BMI and severely obese cohorts in categorical RVF (p = 0.561), with 76% of patients having normal RVF.

**Table 1:** Baseline characteristics

Characteristics		Normal (n = 50)	Severe obesity (n = 50)	Total (n = 100)	p-value
Age, mean (SD) (min, max), years		54.5 (18.7) (22, 92)	47.2 (13.3) (20, 73)	50.8 (16.6) (20, 92)	0.027
Gender	Male	22 (44%)	29 (58%)	51 (51%)	0.161
	Female	28 (56%)	21 (42%)	49 (49%)	
Race	Black/Other	13 (26%)	18 (36%)	31 (31%)	0.279
	Caucasian	37 (74%)	32 (64%)	69 (69%)	
COPD status at time of TTE		7 (14%)	10 (20%)	17 (17%)	0.424
Height, mean (SD) (min, max), inches		66.7 (4.6) (59, 76)	68.3 (3) (60, 72)	67.5 (4) (59, 76)	0.043
Weight, mean (SD) (min, max), pounds		141.3 (23) (100.5, 186.1)	332.4 (29.2) (300, 418)	236.9 (99.5) (100.5, 418)	< 0.001
BMI, mean (SD) (min, max), kg/m^2^		22.2 (1.7) (19.4, 24.8)	50.3 (5.9) (40.7, 66.8)	36.2 (14.8) (19.4, 66.8)	< 0.001
Time between MRI and TTE, mean (SD) (min, max), days		84.9 (89.1) (0, 316)	81.4 (87.4) (0, 325)	83.2 (87.8) (0, 325)	0.845
TTE contrast use		10 (20%)	32 (64%)	42 (42%)	< 0.001
MRI contrast use		47 (94%)	48 (96%)	95 (95%)	0.648
MRI indication	Heart failure	38 (76%)	41 (82%)	79 (79%)	0.303
	Congenital	1 (2%)	4 (8%)	5 (5%)	
	Pericardial	1 (2%)	1 (2%)	2 (2%)	
	Ischemia	5 (10%)	1 (2%)	6 (6%)	
	Cardiac mass	1 (2%)	0 (0%)	1 (1%)	
	Valvular disease	4 (8%)	3 (6%)	7 (7%)	
TTE indication	Heart failure	17 (34%)	33 (66%)	50 (50%)	0.001
	Chest pain	2 (4%)	2 (4%)	4 (4%)	
	Myocardial infarction	6 (12%)	0 (0%)	6 (6%)	
	Pulmonary embolism	1 (2%)	0 (0%)	1 (1%)	
	Arrhythmia	9 (18%)	7 (14%)	16 (16%)	
	Cardiac arrest	2 (4%)	0 (0%)	2 (2%)	
	Congenital	1 (2%)	4 (8%)	5 (5%)	
	Cardiac mass	2 (4%)	0 (0%)	2 (2%)	
	Pre-operative evaluation	3 (6%)	1 (2%)	4 (4%)	
	Stroke	2 (4%)	0 (0%)	2 (2%)	
	Syncope	1 (2%)	2 (4%)	3 (3%)	
	Valvular disease	4 (8%)	1 (2%)	5 (5%)	
MRI LV ejection fraction, mean (SD) (min, max), %		45.5 (14.5) (10, 66)	47.4 (17.6) (7, 76)	46.5 (16.1) (7, 76)	0.558
MRI RV ejection fraction, mean (SD) (min, max), %		52 (11.5) (17, 68)	47.8 (12.5) (20, 64)	50.4 (12) (17, 68)	0.127
MRI RV function	Normal	35 (70%)	31 (63%)	66 (67%)	0.607
	Mild	8 (16%)	6 (12%)	14 (14%)	
	Moderate	4 (8%)	7 (14%)	11 (11%)	
	Severe	3 (6%)	5 (10%)	8 (8%)	
TTE LV ejection fraction, mean (SD) (min, max), %		46 (15.3) (10, 67.5)	49.4 (16.3) (10, 78)	47.7 (15.8) (10, 78)	0.283
TTE RV function	Normal	39 (78%)	37 (74%)	76 (76%)	0.561
	Mild	7 (14%)	7 (14%)	14 (14%)	
	Moderate	4 (8%)	4 (8%)	8 (8%)	
	Severe	0 (0%)	2 (4%)	2 (2%)	

*COPD* chronic obstructive pulmonary disease, *BMI* body mass index, *MRI* magnetic resonance imaging, *TTE* transthoracic echocardiography, *LV* left ventricle, *RV* right ventricle

### MRI and TTE image quality score comparison between cohorts

**Fig. 3:**
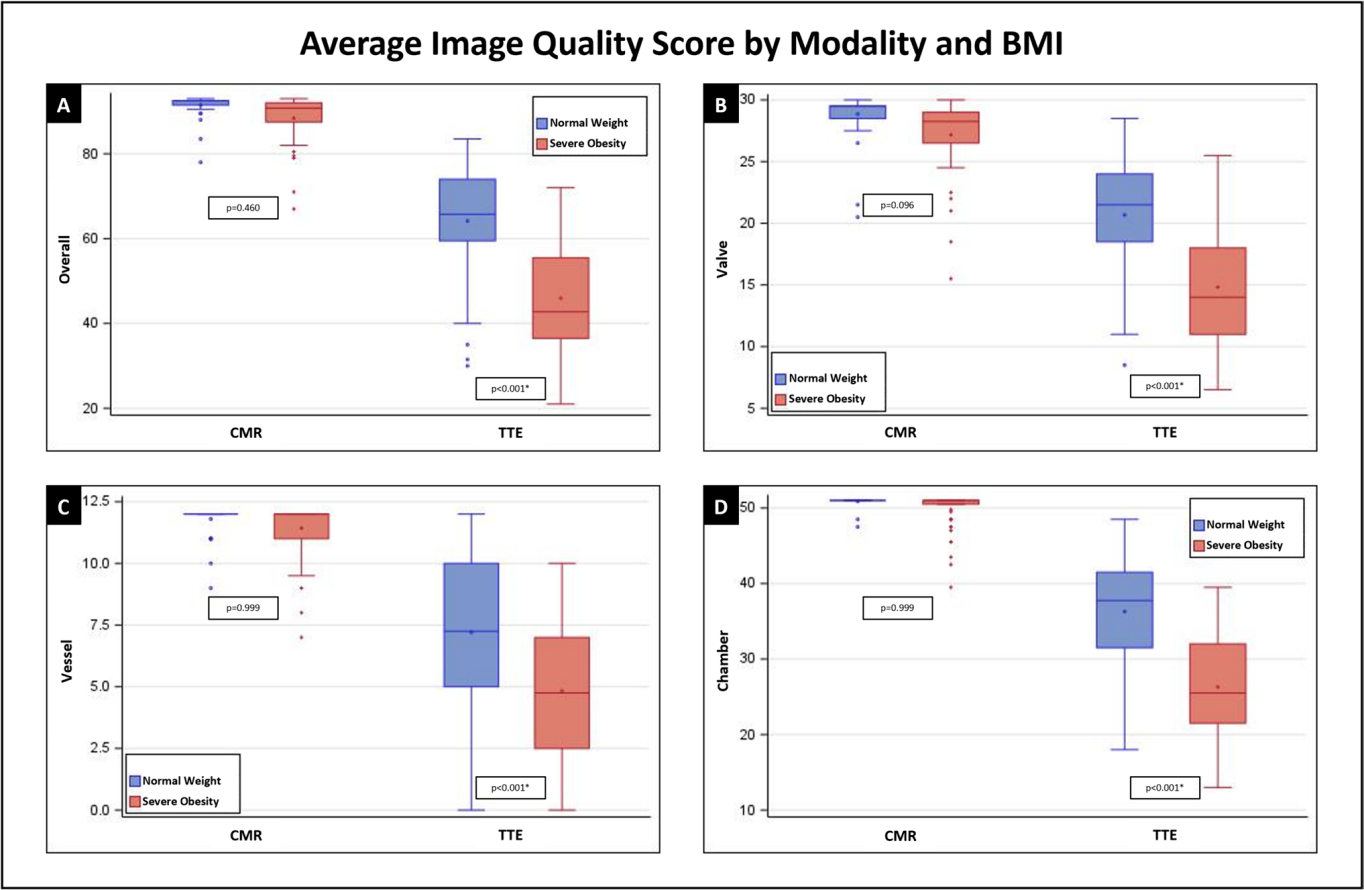
Average image quality score by modality and BMI. **A** shows overall image quality scores, **B** shows valve image quality scores, **C** shows vessel image quality scores, while Panel D shows chamber image quality scores

**Fig. 4:**
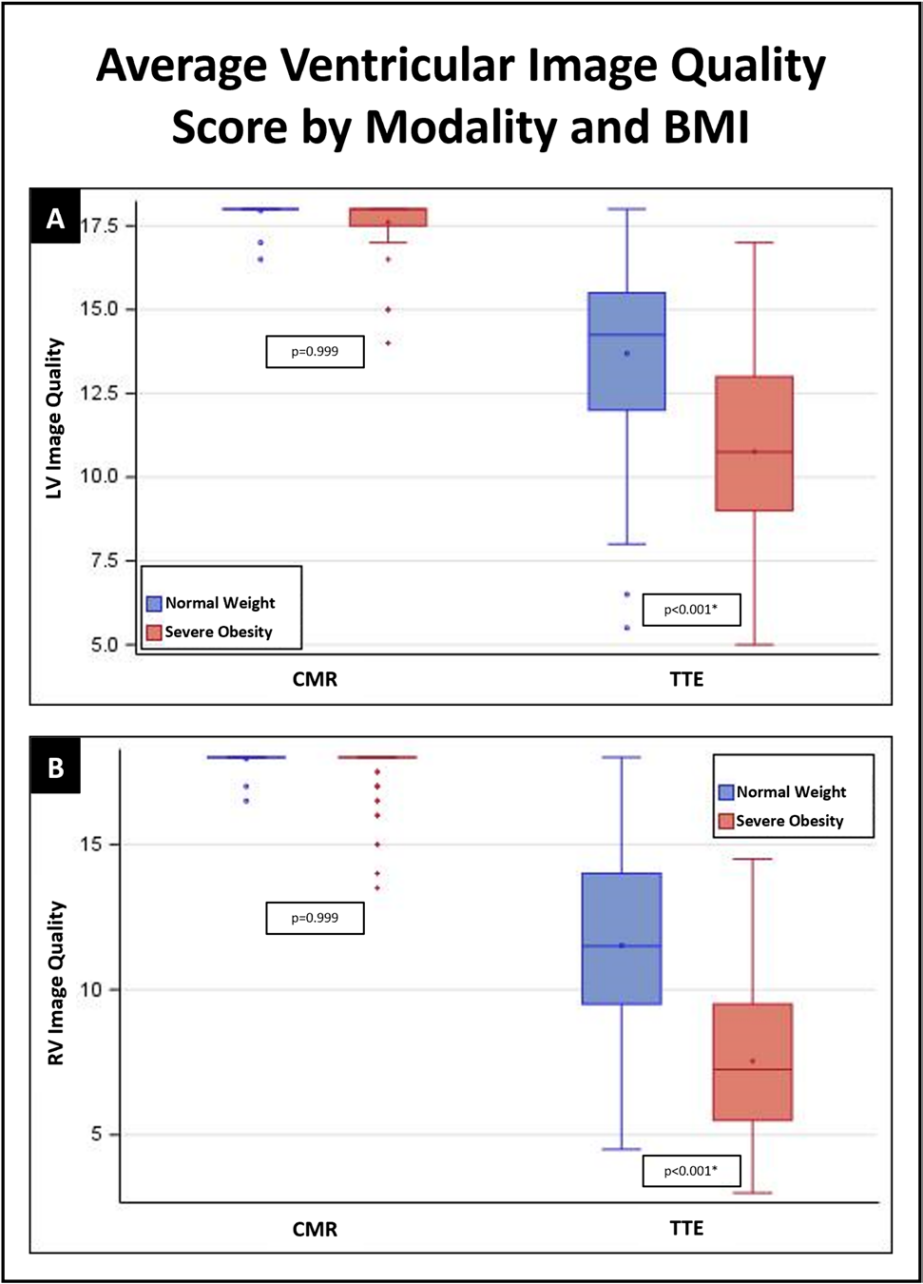
Average ventricular image quality score by modality and BMI. **A** shows left ventricular image quality scores while **B** shows right ventricular image quality scores

Out of a possible 93 points, mean overall image quality score for MRI was similar between the normal BMI (91.5 ± 2.5) and severely obese (88.4 ± 5.5) cohorts; least square (LS) mean difference 3.1, p = 0.460 (Table 2, Fig. 3). Subsection MRI scores including chambers, valves, vessels, and LV and RV demonstrated similarly high and preserved image quality scores between normal BMI and severely obese cohorts (Table 2, Figs. 3, 4).

**Table 2:** Image quality scores by imaging modality and weight class

	Normal (n = 50)	Severe obesity (n = 50)	Total (n = 100)	Least square mean differences (SE)	Adjusted p-value
*Cardiac MRI*					
Total score average, mean (SD) (min, max)	91.5 (2.5) (78, 93)	88.4 (5.5) (67, 93)	90 (4.6) (67, 93)	3.13 (2.0)	0.460
Valve average, mean (SD) (min, max)	28.9 (1.8) (20.5, 30)	27.2 (3) (15.5, 30)	28 (2.6) (15.5, 30)	1.68 (0.7)	0.096
Vessel, mean (SD) (min, max)	11.8 (0.6) (9, 12)	11.4 (1.1) (7, 12)	11.6 (0.9) (7, 12)	0.37 (0.4)	0.999
Chamber, mean (SD) (min, max)	50.9 (0.6) (47.5, 51)	49.8 (2.5) (39.5, 51)	50.3 (1.9) (39.5, 51)	1.08 (1.1)	0.999
LV without mass, mean (SD) (min, max)	18 (0.3) (16.5, 18)	17.6 (0.9) (14, 18)	17.8 (0.7) (14, 18)	0.34 (0.4)	0.999
RV, mean (SD) (min, max)	18 (0.3) (16.5, 18)	17.5 (1.1) (13.5, 18)	17.7 (0.8) (13.5, 18)	0.45 (0.4)	0.999
*TTE*					
Total score average, mean (SD) (min, max)	64.2 (13.6) (30, 83.5)	46 (12.9) (21, 72)	55.1 (16) (21, 83.5)	18.22 (2.0)	< 0.001
Valve average, mean (SD) (min, max)	20.7 (4.3) (8.5, 28.5)	14.8 (4.8) (6.5, 25.5)	17.7 (5.4) (6.5, 28.5)	5.85 (0.7)	< 0.001
Vessel, mean (SD) (min, max)	7.2 (3.1) (0, 12)	4.8 (2.9) (0, 10)	6 (3.2) (0, 12)	2.38 (0.4)	< 0.001
Chamber, mean (SD) (min, max)	36.3 (7.7) (18, 48.5)	26.3 (6.7) (13, 39.5)	31.3 (8.8) (13, 48.5)	9.99 (1.1)	< 0.001
LV without mass, mean (SD) (min, max)	13.7 (2.8) (5.5, 18)	10.8 (2.6) (5, 17)	12.2 (3.1) (5, 18)	2.93 (0.4)	< 0.001
RV, mean (SD) (min, max)	11.5 (3.3) (4.5, 18)	7.5 (2.8) (3, 14.5)	9.5 (3.7) (3, 18)	3.98 (0.4)	< 0.001

Bonferroni correction was employed to reduce Type I error due to four comparisons (two comparisons within modality and two comparisons within BMI)

*MRI* magnetic resonance imaging, *TTE* transthoracic echocardiography, *LV* left ventricle, *RV* right ventricle

Mean overall total image quality score for TTE was significantly higher in the normal BMI cohort (64.2 ± 13.6) compared with the severely obese cohort (46.0 ± 12.9); LS mean difference 18.2, p < 0.001 (Table 2, Fig. 3). Subsection TTE scores including chambers, valves, and vessels in addition to LV and RV focused image quality subsets were all significantly lower in the severely obese cohort (Table 2, Figs. 3, 4). Differences in TTE image quality scores between the cohorts were not explained by chronic obstructive pulmonary disease status (p = 0.424).

Overall image quality was higher in MRI than TTE in the normal BMI cohort (91.5 ± 2.5 vs 64.2 ± 13.6; LS mean difference 27.4, p < 0.001), and this discrepancy was even greater in the severely obese cohort (88.4 ± 5.5 vs 46.0 ± 12.9; LS mean difference 42.4, p < 0.001). Similar findings were noted in the scoresheet subsections, and LV and RV subsets (Figs. 3, 4). The decrease in overall image quality scores observed from the normal BMI to the severely obese cohort was greater in TTE (39.6% drop) compared to MRI (3.5% drop).

**Fig. 5:**
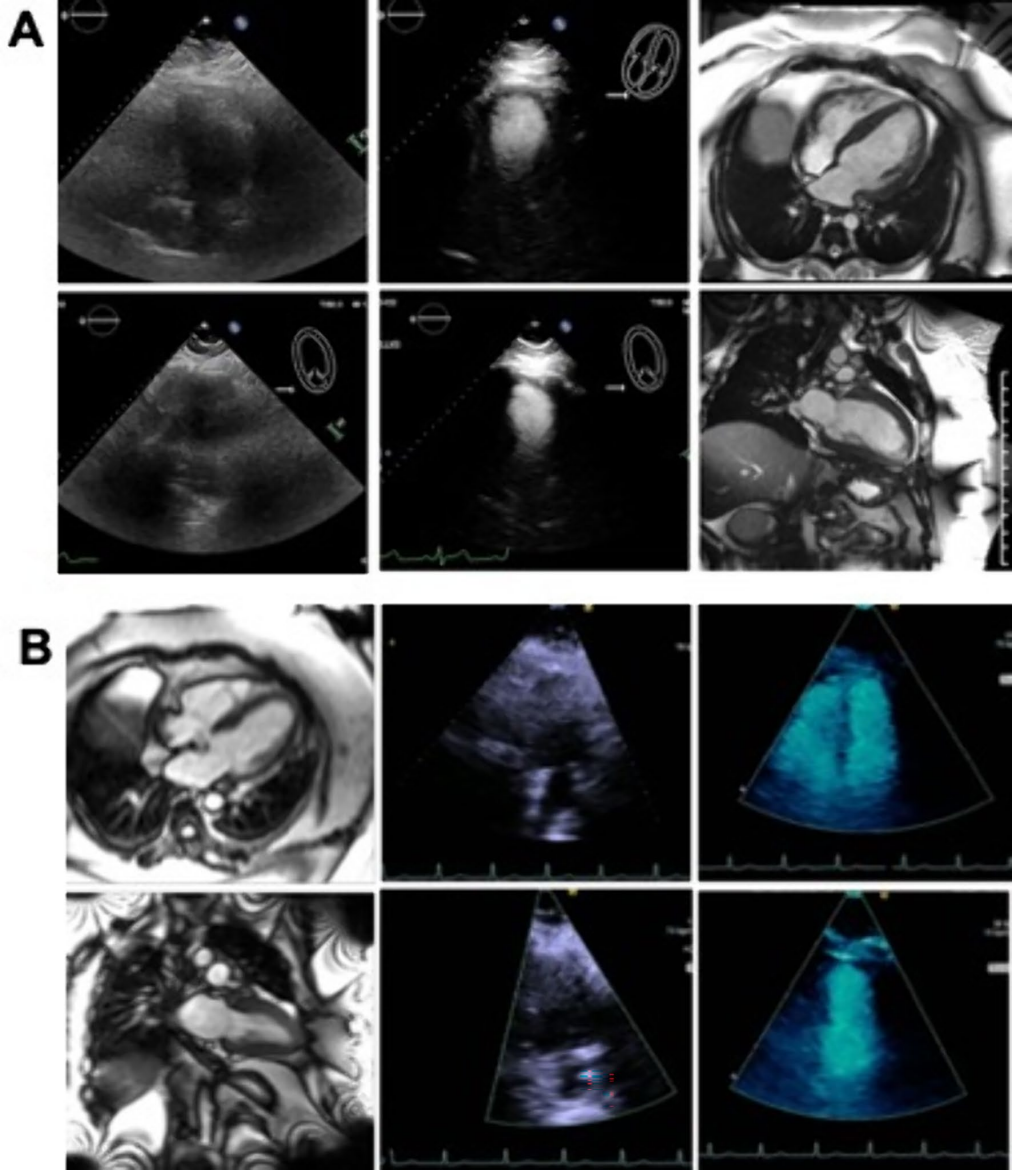
**A** Example of low overall echocardiography image quality score. Top (left to right) – 4 chamber TTE views without, with microbubble contrast, MRI steady state free precession cine. Bottom: 2 chamber TTE views without, with microbubble contrast, MRI steady state free precession cine. Overall image quality score: TTE – 21; MRI – 91. *TTE* transthoracic echocardiography, *MRI* magnetic resonance imaging. BMI 49.2. **B** Example of low overall cardiac magnetic resonance image quality score**.** Top (left to right) – 4 chamber MRI steady state free precession cine, TTE view without, with microbubble contrast. Bottom: 2 chamber MRI steady state free precession cine, TTE view without, with microbubble contrast. Overall image quality score: MRI – 71; TTE – 43.5. *TTE* transthoracic echocardiography, *MRI *magnetic resonance imaging. BMI = 56.5

Additional analysis was performed on the subset of patients who received microbubble contrast for TTE. In the normal BMI cohort, the mean overall TTE image quality score for patients not receiving contrast was not significantly different from those receiving contrast (65.7 ± 2.0 vs 58.0 ± 4.1, respectively; LS mean difference 7.8 ± 4.6, p = 0.184). In the severely obese cohort, the mean overall TTE image quality score for patients not receiving contrast was also not significantly different from those receiving contrast (50.9 ± 3.0 vs 43.2 ± 2.3, respectively; LS mean difference 7.7 ± 3.8, p = 0.093). Representative example MRI and TTE images with and without contrast demonstrating cases with both low TTE image quality scores and low MRI image quality scores can be seen in Fig. 5A and B.

### Agreement between cardiac MRI and TTE ventricular function assessment

**Fig. 6:**
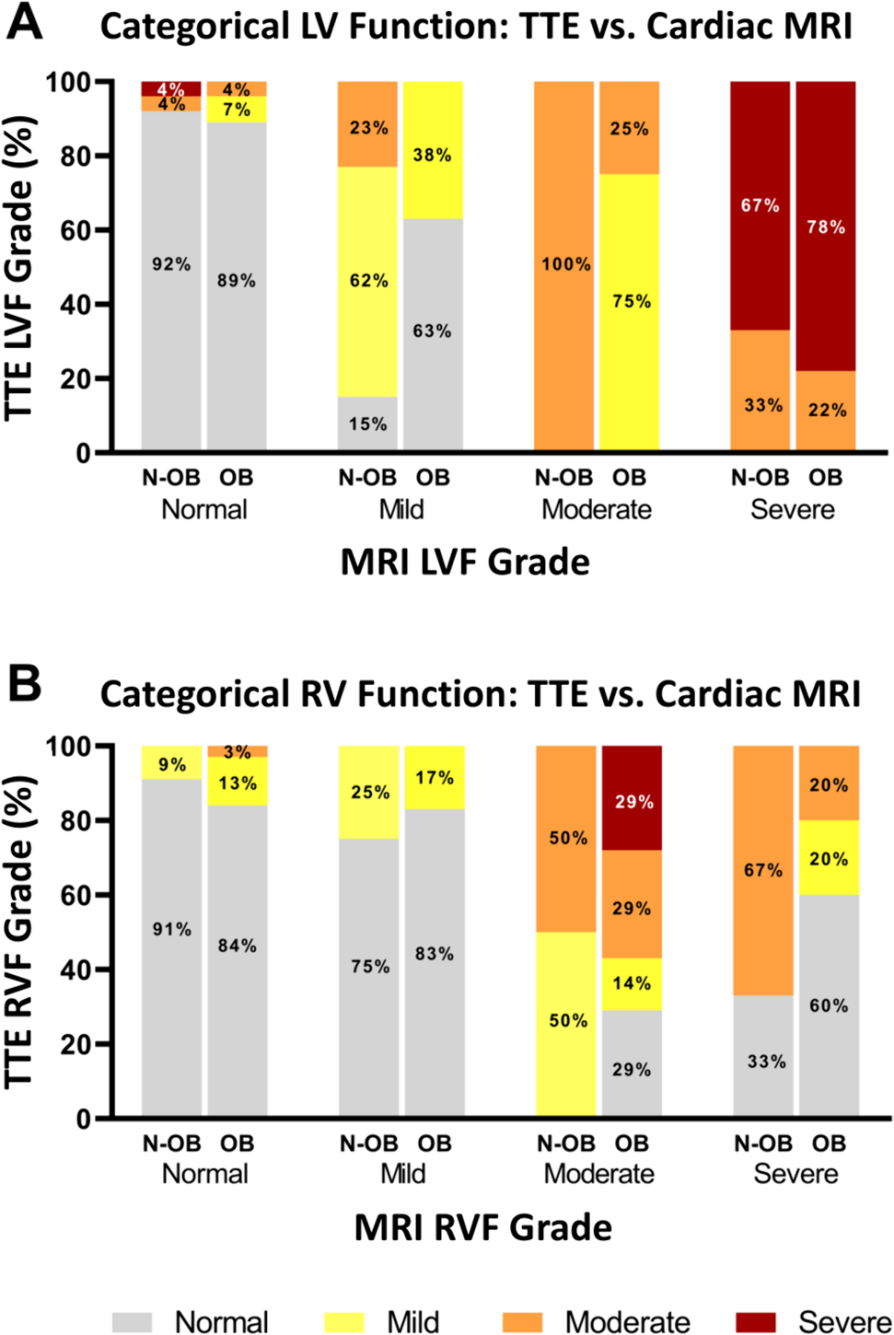
TTE and cardiac MRI categorical ventricular function agreement by BMI class. **A** Agreement between cardiac MRI and TTE LV function categories by BMI class. **B** Agreement between cardiac MRI and TTE RV function categories. By BMI class. *BMI* body mass index, *N-OB* non-obese, *OB* obese.

Comparing categorical LVF assessment, there was substantial agreement between cardiac MRI and TTE for the normal BMI cohort (kappa = 0.70, p < 0.001), but only moderate agreement in the severely obese cohort (kappa = 0.53, p < 0.001). There was a higher percentage of patients in the normal BMI cohort in whom MRI and TTE LVF category matched, compared with the obese BMI cohort (80% vs 72% of MRI-TTE pairs with agreement, p < 0.001) (Table 3A, Fig. 6A). There was fair agreement between MRI and TTE in the categorical assessment of RVF for the normal BMI cohort (kappa = 0.34, p < 0.001), but there was no agreement in the severely obese cohort (kappa = 0.18, p = 0.004). There was a higher percentage of patients in whom MRI and TTE RVF category matched in the normal BMI cohort compared with the severely obese BMI cohort (72% vs 58% of MRI-TTE pairs with agreement, p < 0.001) (Table 3B, Fig. 6B). Among 53 patients with LVF < 50% determined by either or both modalities, there were 16 patients with discrepant MRI and TTE LVF around the specified < 50% or < 35% cutoffs, of which only five (9%) could possibly be explained by a clinical status change occurring between the two imaging study dates. Scatter plots of differences in LVF and RVF categories across time did not demonstrate an identifiable pattern (Supplemental Data).

**Table 3:** TTE and cardiac MRI (A) left ventricular function agreement by weight class; (B) right ventricular function agreement by weight class

		TTE						TTE			
		Normal	Mild	Moderate	Severe			Normal	Mild	Moderate	Severe
(A)						(B)					
Normal BMI^a^						Normal BMI^c^					
MRI	Normal	22	0	1	1	MRI	Normal	32	3	0	0
	Mild	2	8	3	0		Mild	6	2	0	0
	Moderate	0	0	4	0		Moderate	0	2	2	0
	Severe	0	0	3	6		Severe	1	0	2	0
Severe obesity^b^						Severe obesity^d^					
MRI	Normal	25	2	1	0	MRI	Normal	26	4	1	0
	Mild	5	3	0	0		Mild	5	1	0	0
	Moderate	0	3	1	0		Moderate	2	1	2	2
	Severe	0	0	2	7		Severe	3	1	1	0

Kappa interpretation					
Kappa statistics	Agreement				
0–0.20	None to slight				
0.21–0.40	Fair				
0.41–0.60	Moderate				
0.61–0.80	Substantial				
0.81–1.00	Almost perfect				

*BMI* body mass index, *MRI* magnetic resonance imaging, *TTE* echocardiogram

^a^Kappa: 0.70 (0.55 0.85), p < 0.001

^b^Kappa: 0.53 (0.34 0.72), p < 0.001

^c^Kappa: 0.34 (0.11 0.56), p < 0.001

^d^Kappa: 0.18 (0.009 0.38), p = 0.004

### MRI tissue characterization

MRI-derived myocardial T1 and T2 mapping, and LGE were performed in 70%, 66%, and 95% of patients, respectively. Median T1, T2, and LGE image quality scores were high. T1 and LGE image quality was similar between the cohorts (p = 0.289 and p = 0.090, respectively), while T2 mapping image quality scored higher in the normal BMI cohort, p = 0.030 (Table 4).

**Table 4:** Cardiac MRI tissue characterization

Variable	Normal (n = 50)	Severe obesity (n = 50)	Total (n = 100)	p-value
MRI T1, N, median [IQR] (min, max)	43, 3 [3] (1.5, 3)	27, 3 [3] (2.5, 3)	70, 3 [3] (1.5, 3)	0.289
MRI T2, N, median [IQR] (min, max)	39, 3 [3] (0.5, 3)	27, 2.5 [3] (2, 3)	66, 3 [3] (0.5, 3)	0.030
MRI LGE, N, median [IQR] (min, max)	47, 3 [3] (1.5, 3)	48, 3 [3] (2.5, 3)	95, 3 [3] (1.5, 3)	0.09

*MRI* magnetic resonance imaging, *LGE* late gadolinium enhancement

## Discussion

Our objectives were to determine the effect of severe obesity on image quality for cardiac MRI and TTE, and to assess ventricular function agreement between modalities. When compared with patients with normal BMI, image quality for patients with severe obesity remained high and preserved for cardiac MRI but was negatively impacted for TTE. Furthermore, categorical agreement in LV and RV function between cardiac MRI and TTE was worse in the severely obese cohort compared with the normal weight cohort, which may lead to misdiagnosis or inappropriate treatment. These findings highlight the significance of choosing the appropriate imaging modality for clinical assessment and underscore the link between image quality, diagnostic assessment, and subsequent treatment decisions.

This study introduced a novel scoring system to evaluate and compared MRI and TTE image quality in a semi-quantitative manner. The scoring focused on criteria assessable by both modalities, such as ventricular morphology and function, excluding modality-specific criteria like myocardial fibrosis. While prior studies have examined image quality within populations [22] or modalities [23], our approach compared image quality between populations (normal BMI vs. severely obese) and across modalities (MRI vs. TTE). Image quality across all modalities can be compromised in patients with severe obesity [8, 24, 25], and while MRI and TTE are routinely used to evaluate these patients, a head-to-head comparison of the impact of severe obesity on both MRI and TTE had not been previously investigated.

Our finding that TTE image quality significantly degrades in patients with severe obesity is in agreement with previous studies [26, 27]. Increased fat mass causes signal attenuation, reducing the capacity of TTE to assess cardiac morphology, function, and flow [28, 29]. The use of microbubble contrast agent can help to maintain TTE image quality in patients with obesity, although adding to the complexity and cost of the exam [7, 15]. Ellenberger et al. recently reported echo contrast use of 23% of patients with normal BMI and 71% of patients with severe obesity, comparable to the 20% and 64% rates of contrast usage, respectively in our cohorts [30]. We found that patients receiving microbubble contrast in either normal BMI or severely obese cohorts had similar image quality scores as those not receiving contrast. Although contrast may enhance the LV endocardial border, it does not typically improve assessment of valves, vessels, or atria. As supported by Ellenberger et al. and our findings of similar TTE image quality scores with and without contrast, we speculate that microbubble contrast is often used when pre-contrast image quality is severely compromised and does not necessarily improve image quality substantially when pre-contrast quality is poor.

Consistent with the assumption that MRI is the modality least sensitive to image quality degradation caused by obesity [31], we observed no significant difference in cardiac MRI image quality between normal BMI and obese cohorts. Beyond the function and flow measurements afforded by TTE, MRI provides myocardial tissue characterization and the ability to evaluate scar, fibrosis, and edema. In our study, T1 mapping and LGE demonstrated consistent image quality between BMI cohorts, indicating feasibility regardless of body habitus. However, severe obesity poses challenges for MRI as well, including bore size and patient table weight limitations, increased burn risk caused by skin folds, and increased rates of claustrophobia [8]. Lower field, open MRI scanners have been available for some time, offering an alternative that can accommodate patients with severe obesity and/or claustrophobia. While cardiac MRI has been demonstrated on these systems [32, 33], an open magnet configuration can require compromises in overall system performance. The recent introduction of low-field MRI scanners with conventional magnet design but larger bore diameter and higher table weight limits may help expand MRI access to patients with severe obesity [34]. Additionally, low-field systems are significantly less costly to acquire, install, and operate than standard 1.5 T or 3 T scanners, and recent reports are demonstrating good cardiac image quality [35].

When examining differences between cardiac MRI and TTE imaging quality, we found that MRI image quality was superior to TTE in both normal BMI and severely obese cohorts, and this difference was more pronounced in the severely obese cohort. This observed overall image quality effect was also seen in each pre-specified subcategory (chambers, valves, and vessels), suggesting that the overall score difference was not skewed by the effect of a single subcategory. A study conducted by Kanagala et al. also found image quality differences between MRI and TTE in patients with heart failure [22]; the authors attributed this in part to the effect on TTE image quality of common comorbidities including obesity [22, 35].

Cardiac MRI is widely accepted as the reference standard for cardiac morphology and function, and we found discrepancies in MRI and TTE measures of left and right ventricular function with poorer agreement between TTE and MRI in patients with severe obesity compared with those with normal BMI. LVF was misclassified by TTE in approximately one-third of patients with severe obesity, and RVF misclassified in nearly one-half. Notably, RVF by TTE has previously demonstrated varying correlation with MRI [13, 36], and our findings also reflect this real-world variability with no agreement in RVF in patients with severe obesity. Review of the 53 patients with LVF < 50%, only 9% of these had clinical status changes (one arrest, three restoration of sinus rhythm, and one due to medical treatment of heart failure). The remaining LVF discrepancies could not be explained by interval clinical events suggesting the differences in ventricular function may be better attributable to image quality. Despite a majority of patients in both cohorts having normal cardiac function, we demonstrated a significant effect of severe obesity on categorical ventricular function assessment. We speculate that a larger cohort with abnormal cardiac function would have more dramatic observations.

Our study has several limitations. The retrospective design introduces potential referral biases, although no MRI studies were included with an indication of non-diagnostic TTE. We were unable to account for patients not referred to MRI due to claustrophobia or an inability to fit into the scanner bore, and MRI studies that were not completed due to patient discomfort or claustrophobia. Additionally, the retrospective nature of the study and non-contemporaneous scans introduce the possibility of circumstantial events between scans that could impact cardiac function and image quality. Two-thirds of patients had their MRI and TTE performed within 90 days apart, and given that the time differences between TTE and MRI were not statistically different between the normal BMI and severely obese cohorts, we expect that any potential time-related biases would have been equivalent (Supplemental Data). While our novel image quality scoring system was not previously validated, the scores confirmed the quality degradation due to severe obesity by TTE but preserved in cardiac MRI. Future prospective studies employing simultaneous MRI and TTE assessments with standardized LVF and RVF quantification methods would help mitigate any biases introduced by the time between exams.

Our cohorts comprised only normal weight and severely obese patients and did not include those falling into overweight to moderately obese categories (25 < BMI < 40). Therefore, we cannot draw any conclusions regarding MRI or TTE image quality in these intermediate groups. Finally, all MRI scans were done at 1.5 T. Cardiac MRI is also commonly performed at 3 T, which offers higher signal-to-noise ratio that can be used to increase spatial resolution or shorten scan time. However, artifacts caused by field inhomogeneity are worse at higher field, thus it is unclear how our results would translate to 3 T MRI.

In conclusion, cardiac MRI may provide more reliable diagnostic information than TTE in patients with severe obesity, suggesting cardiac MRI could be used as the first-line modality choice for CVD evaluation in this population. Cardiac MRI image quality was preserved in patients with severe obesity, whereas TTE showed a significant quality degradation in this group. Contrast-enhanced TTE did not demonstrate superior image quality compared to non-contrast exams in either patient cohort. Furthermore, there was worse agreement between MRI and TTE in the categorical assessment of LVF and RVF in patients with severe obesity. With a high and increasing prevalence of severe obesity [2], high quality and accurate imaging techniques are necessary to support best clinical practice.

### Supplementary Information

Below is the link to the electronic supplementary material.

Supplementary file1 (DOCX 216 KB)

## Abbreviations

ACC: American College of Cardiology
COCATS: Core Cardiology Training Symposium
CVD: Cardiovascular disease
LVF: Left ventricular function
RVF: Right ventricular function
LV: Left ventricle
RV: Right ventricle
SNR: Signal–noise ratio
OSUWMC: The Ohio State University Wexner Medical Center
LGE: Late gadolinium enhancement
