# Supplementary material for: The impact of severe obesity on image quality and ventricular function assessment in echocardiography and cardiac MRI
Source: Int J Cardiovasc Imaging. 2024 Apr 16;40(5):1081–94. doi: 10.1007/s10554-024-03078-y (PMC11147879; doi:10.1007/s10554-024-03078-y)

**Supplemental Data**

Categorical differences between LV and RV Function plotted against Time between Cardiac MRI and Echocardiogram


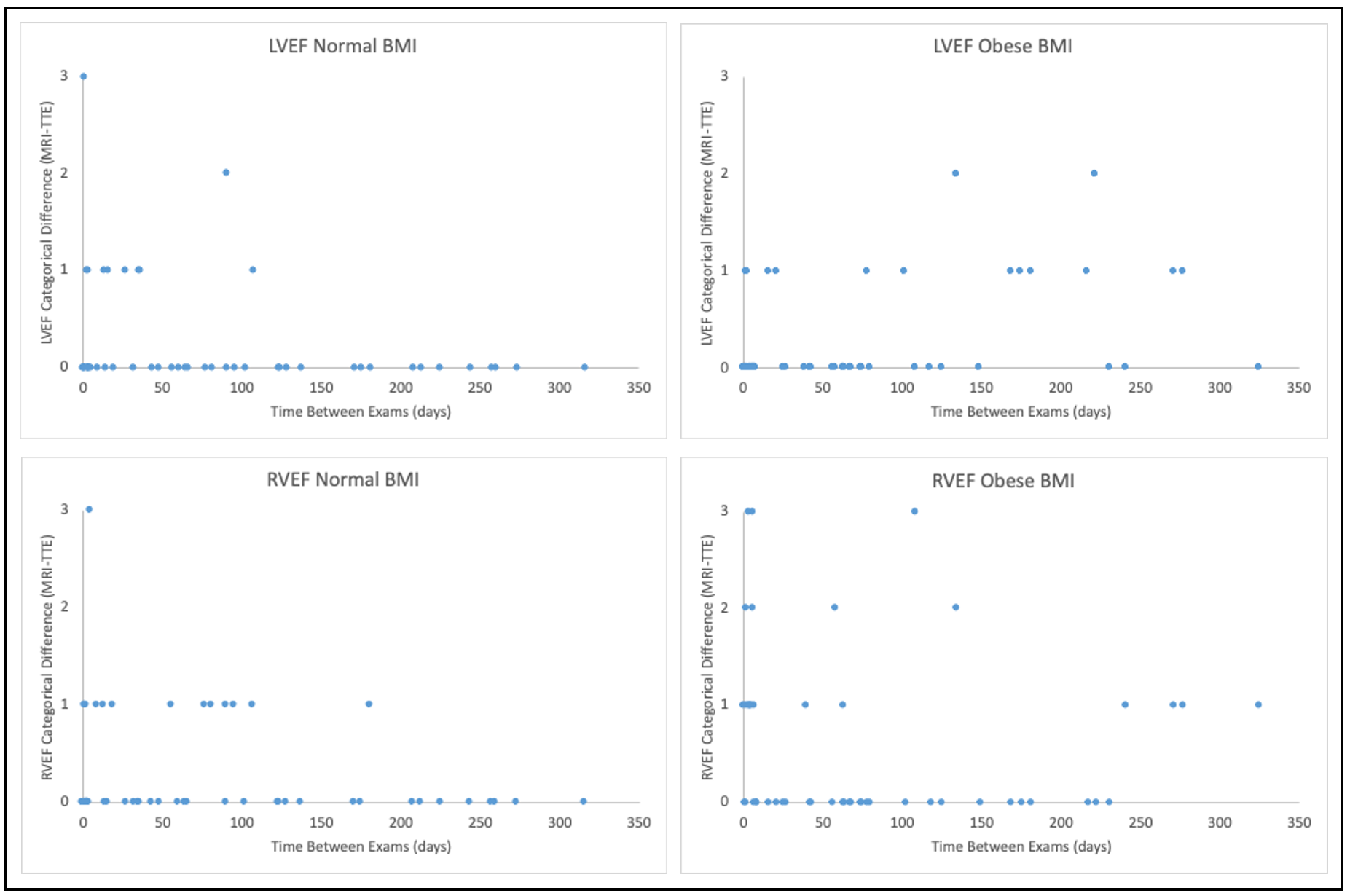

Supplement: Supplementary file 1 — Supplementary file1 (DOCX 216 KB) [file 10554_2024_3078_MOESM1_ESM.docx]
